# Comparative Circulating microRNA Profiling in Dogs with Pyometra and Other Inflammatory Diseases

**DOI:** 10.3390/vetsci13040387

**Published:** 2026-04-16

**Authors:** Myeong-Seon Jeong, Gyo-Bin Choo, Bum-Kyu Choi, Mun-Ik Lee, Ju-Hyun An

**Affiliations:** 1 Department of Veterinary Emergency and Critical Care Medicine, College of Veterinary Medicine, Kangwon National University, Chuncheon 24341, Republic of Korea; audtjs0805@naver.com (M.-S.J.); ckyobin@naver.com (G.-B.C.); 202011254@kangwon.ac.kr (B.-K.C.); 2 Reborn Animal Medical Center, Chuncheon 24380, Republic of Korea; dlansdlr9907@gmail.com

**Keywords:** canine disease, circulating microRNA, gene expression profiling, plasma biomarkers, small RNA sequencing, systemic inflammation

## Abstract

Circulating microRNAs (miRNAs) regulate inflammatory responses and have emerged as potential minimally invasive biomarkers of disease. However, comprehensive profiling of circulating miRNAs in naturally occurring inflammatory conditions in dogs remains limited. This study aimed to characterize circulating miRNA expression profiles using small RNA sequencing in plasma samples from dogs with pyometra, non-reproductive inflammatory diseases, and healthy controls to identify shared and disease-related miRNA alterations. Global expression patterns, including heatmap and principal component analysis (PCA), demonstrated separation between healthy controls and diseased dogs. Based on DESeq2 analysis (adjusted *p*-value < 0.05), 39 circulating miRNAs were commonly altered in both pyometra and non-reproductive inflammatory diseases compared with healthy controls. A total of 83 miRNAs were differentially expressed in the pyometra group, whereas 4 miRNAs were differentially expressed in the non-reproductive inflammatory disease group. Direct comparison between the two disease groups further identified three circulating miRNAs—cfa-miR-885, cfa-miR-599, and cfa-miR-122—as significantly differentially expressed. These findings suggest that circulating miRNA profiles in dogs with pyometra reflect both systemic inflammation and condition-related molecular variation. Further studies with larger cohorts are warranted to validate the clinical relevance of these candidate circulating miRNAs.

## Simple Summary

Pyometra is a common and potentially life-threatening uterine infection in intact female dogs and is often associated with systemic inflammation. Circulating microRNAs (miRNAs) are small regulatory molecules found in blood that can reflect physiological and pathological changes in the body and may serve as minimally invasive biomarkers of disease. However, global profiling of circulating miRNAs in naturally occurring inflammatory diseases in dogs remains limited. In this study, we analyzed circulating miRNA expression profiles in dogs with pyometra and dogs with non-reproductive inflammatory diseases using small RNA sequencing and compared them with healthy dogs. We identified several circulating miRNAs that were altered in both disease groups, suggesting that these miRNAs may reflect systemic inflammatory responses. In addition, some miRNAs were differentially expressed in dogs with pyometra, indicating potential disease-associated molecular differences. These findings provide preliminary insights into circulating miRNA changes associated with inflammatory conditions in dogs and suggest that plasma miRNAs may help improve the understanding of systemic inflammation and disease-related molecular variation. Further studies with larger populations are needed to validate the diagnostic or clinical relevance of these candidate miRNAs.

## 1. Introduction

Inflammation is a fundamental immune response to harmful stimuli. However, when inflammatory responses become excessive or poorly regulated, they may progress to systemic inflammatory response syndrome (SIRS), which can lead to microvascular injury, hemodynamic instability, and ultimately multiple organ dysfunction syndrome (MODS) [1].

Pyometra is one of the most common reproductive diseases in intact female dogs. In the early stages, clinical signs are often subtle and non-specific, which can delay diagnosis. As the disease progresses, the inflammatory process may extend beyond the uterus and lead to systemic complications such as SIRS, sepsis, and organ dysfunction. Although ultrasonography is widely used for diagnosis, it primarily evaluates structural uterine changes and provides limited information regarding disease severity or the systemic inflammatory status of affected dogs [2]. These limitations highlight the need to identify molecular regulators and biomarkers that better reflect the dynamic processes of systemic inflammation.

MicroRNAs (miRNAs) are small non-coding RNAs, approximately 18–25 nucleotides in length, that regulate gene expression at the post-transcriptional level through sequence-specific interactions with messenger RNAs (mRNAs) [3]. By modulating mRNA stability or translation, miRNAs influence numerous biological pathways, including immune regulation and inflammatory signaling. In human medicine, altered miRNA expression has been associated with a wide range of pathological conditions, including cancer, inflammatory disorders, metabolic diseases, and autoimmune conditions [4]. Because miRNAs regulate multiple stages of the inflammatory response—such as initiation, amplification, and resolution—changes in their expression patterns may reflect dysregulation of immune pathways and offer potential utility as diagnostic biomarkers or therapeutic targets [5].

Circulating cell-free miRNAs can be detected in serum and plasma and remain stable even in RNase-rich environments. Although the mechanisms underlying the release of miRNAs into circulation are not fully understood, activated immune cells and damaged tissues are considered likely sources. Compared with tissue-based diagnostics, circulating miRNAs offer the advantage of minimally invasive sampling and allow repeated assessment over time. Recent human studies have demonstrated the potential clinical value of circulating miRNAs in inflammatory diseases such as inflammatory bowel disease, rheumatoid arthritis, and asthma [5].

Although miRNA research in veterinary medicine is less extensive than in human medicine, growing evidence suggests that many miRNA regulatory pathways are conserved across species [5,6]. Altered miRNA expression has been reported in various canine diseases, including infectious diseases such as Leishmania spp., Toxocara canis, canine influenza virus, enterovirus, and canine alpha-herpesvirus 1 infections [6,7]. Dysregulated miRNA expression has also been described in non-infectious inflammatory disorders, including atopic dermatitis, hepatitis, inflammatory bowel disease, meningoencephalomyelitis, otitis externa, peri-implantitis, and prostatitis [6]. In addition, reproductive tract diseases have been associated with distinct miRNA expression patterns, with altered miRNA profiles reported in the ovaries, oviducts, and uteri of dogs with uterine disorders compared with healthy dogs [8].

Despite these advances, variability in sample types and the lack of standardized detection and normalization protocols limit cross-study comparisons. Furthermore, most previous studies of canine inflammatory diseases have relied on candidate-based RT-qPCR approaches, and unbiased global profiling of circulating miRNAs in naturally occurring inflammatory conditions remains limited.

Therefore, the present study investigated circulating miRNA expression profiles in plasma from dogs with pyometra and dogs with non-reproductive inflammatory diseases using small RNA sequencing. Because pyometra involves both systemic inflammation and a hormonally regulated uterine environment, a non-reproductive inflammatory disease group was included to distinguish miRNA alterations associated with general inflammatory responses from those potentially related to pyometra-specific pathophysiology.

## 2. Materials and Methods

### 2.1. Ethical Approval and Study Population

This study was conducted with the approval of the Institutional Animal Care and Use Committee (IACUC) of Kangwon National University (KNU), Republic of Korea (KW-250613-1). Written informed consent was obtained from all dog owners prior to sample collection.

This exploratory comparative study included 15 client-owned dogs divided into three groups: dogs with pyometra (*n* = 5), dogs with non-reproductive inflammatory diseases (*n* = 5), and healthy controls (*n* = 5). Because this study was designed as a pilot exploratory investigation aimed at identifying candidate circulating miRNAs associated with inflammatory conditions, a limited sample size was used to generate preliminary sequencing data for subsequent validation studies.

The pyometra group consisted of intact female dogs diagnosed with naturally occurring pyometra. The diagnosis was established based on compatible clinical signs including vaginal discharge, lethargy, anorexia, polydipsia, and polyuria, together with hematological and biochemical abnormalities and ultrasonographic evidence of uterine enlargement with intraluminal fluid accumulation. Definitive confirmation was obtained following ovariohysterectomy, during which purulent material was identified within the uterine lumen, and cytological examination of the uterine contents revealed the presence of bacteria [9].

The non-reproductive inflammatory disease group consisted of neutered dogs of both sexes presenting with systemic inflammation unrelated to the reproductive tract. Systemic inflammation was defined according to established SIRS criteria and supported by hematologic and biochemical abnormalities consistent with inflammatory status. SIRS was defined as the presence of at least two of the following criteria: body temperature (<38.1 °C or >39.2 °C), heart rate (>120 beats/min), respiratory rate (>20 breaths/min), and leukocyte count (<6 × 10^3^/L, >16 × 10^3^/L, or >3% band forms) [10]. Diagnoses in this group included enteritis (*n* = 2), pancreatitis (*n* = 2), and bite wound–associated soft-tissue infection (*n* = 1).

Healthy control dogs were considered clinically normal based on medical history, physical examination, hematological and biochemical testing, and imaging studies, including thoracic and abdominal radiography and abdominal ultrasonography. None showed clinical or laboratory evidence of systemic disease at the time of enrollment.

Dogs were eligible for inclusion if they weighed less than 10 kg and were older than 3 years of age. To reduce variability associated with body size, the study population was limited to small-breed dogs. Only adult dogs were included to minimize potential age-related variation in circulating miRNA expression. Dogs that had received medications other than routine preventive treatments within the preceding three months were excluded. To minimize potential hormonal influences on circulating miRNA profiles, only neutered dogs were included in the non-reproductive inflammatory disease and healthy control groups. Dogs in the pyometra and non-reproductive inflammatory disease groups were excluded if concurrent inflammatory or reproductive disorders unrelated to the primary diagnosis were present.

### 2.2. Sample Collection, RNA Extraction, and Small RNA Sequencing

Peripheral blood samples were collected at the time of clinical presentation via jugular or cephalic venipuncture into EDTA- and heparin-anticoagulated tubes. EDTA blood samples were used for hematological analysis using an automated hematology analyzer, whereas biochemical measurements were performed using heparinized plasma.

For miRNA profiling, plasma was separated from EDTA-anticoagulated blood by centrifugation. Samples were initially stored at −20 °C for short-term handling and transferred to −80 °C within 24 h for long-term storage. No samples underwent repeated freeze–thaw cycles prior to RNA extraction. Care was taken to minimize hemolysis during sample collection and processing, and no visible hemolysis was observed during sample processing [11].

Total RNA was extracted from 200 μL of plasma using the miRNeasy Serum/Plasma Kit (Qiagen, Hilden, Germany) according to the manufacturer’s instructions. RNA quality was assessed using an Agilent 2100 Bioanalyzer with the RNA 6000 Pico Chip (Agilent Technologies, Amstelveen, The Netherlands), and RNA concentration was measured using a Qubit fluorometer (Thermo Fisher Scientific Inc., Cleveland, OH, USA).

Small RNA libraries were constructed using the NEBNext^®^ Low Bias Small RNA Library Prep Kit (New England BioLabs, Ipswich, MA, USA). Bead-based size selection of small RNA fragments was performed using NEBNext Sample Purification Beads (New England BioLabs, Ipswich, MA, USA, E3429). Library size distribution and concentration were evaluated using an Agilent 4200 TapeStation and a Qubit fluorometer (Thermo Fisher Scientific). Sequencing was performed on an Illumina NextSeq 2000 platform with single-end 75 bp reads (Illumina Inc., San Diego, CA, USA).

Raw sequencing data were evaluated for quality using FastQC [12]. Adapter trimming and quality filtering were performed using BBDuk v38.34 [13]. Clean reads were aligned to mature miRNA reference sequences using Bowtie2 v2.3.5.1 [14], and read counts mapped to mature miRNA sequences were extracted using Bedtools v2.27.1 [15].

Raw read counts were filtered to retain features with at least one read in at least one sample for downstream analysis. To enable robust comparison across samples with varying sequencing depths, differential expression analysis was performed using the DESeq2 package (v1.42.1) [16] in R (v4.3.1). Normalization was performed using the median-of-ratios method to account for differences in sequencing depth and RNA composition. Dispersion estimates were calculated to model gene-wise variability.

### 2.3. Statistical Analysis

Clinical data were expressed as mean ± standard deviation for body weight and as median (range) for age, vital parameters, and clinicopathological measurements. C-reactive protein (CRP) values exceeding the upper measurable limit of the assay (>10 mg/L) were recorded as 10 mg/L for statistical analysis.

Differential expression between groups was assessed using a negative binomial generalized linear model implemented in the DESeq2 package (v1.42.1). Fold changes were estimated, and statistical significance was evaluated using the Wald test. *p*-values were adjusted for multiple testing using the Benjamini–Hochberg false discovery rate (FDR) method. Differentially expressed miRNAs were defined as those with an adjusted *p*-value (padj) < 0.05 and |log2 fold change| ≥ 1.

For visualization, variance-stabilizing transformation (VST) was applied to the count data. Principal component analysis (PCA) and heatmaps were generated using VST-transformed expression values with R (4.3.1). For the heatmap, the top 100 most variable miRNAs were selected based on variance, and row-wise scaling (z-score normalization) was applied to emphasize relative expression patterns across samples. Graphical visualizations, including Venn diagrams, scatter plots, and dot plots, were generated using ExDEGA and ExDEGA GraphicPlus (Ebiogen Inc., Seoul, Korea).

## 3. Results

### 3.1. Patient Characteristics

A total of 15 client-owned small-breed dogs (<10 kg) were included and equally distributed among the control (*n* = 5), non-reproductive inflammatory disease (*n* = 5), and pyometra (*n* = 5) groups. The control group consisted of one castrated male and four spayed females, the non-reproductive inflammatory disease group included three castrated males and two spayed females, and all dogs in the pyometra group were intact females. Median age ranged from 7 to 10 years across groups, and mean body weight ranged from 4.05 to 7.0 kg. Vital parameters, including body temperature, heart rate, respiratory rate, and systolic blood pressure, were generally within reference ranges, although dogs in both disease groups tended to have higher body temperatures than controls. The non-reproductive inflammatory disease group included dogs diagnosed with enteritis (*n* = 2), pancreatitis (*n* = 2), and bite wound–associated soft-tissue infection (*n* = 1). Concurrent conditions included gallbladder sludge (*n* = 1) and myxomatous mitral valve disease (*n* = 1) in the non-reproductive inflammatory disease group, and gallbladder sludge (*n* = 2) in the pyometra group (Table 1).

Clinicopathologic variables for dogs in healthy control, non-reproductive inflammatory disease, and pyometra groups are summarized in Table 2. Compared with healthy controls, dogs in both disease groups showed clinicopathologic changes consistent with systemic inflammation. Total leukocyte counts and neutrophil counts were significantly increased in both disease groups relative to healthy controls. Monocyte counts were also higher in diseased dogs. Hematocrit values were significantly lower in the non-reproductive inflammatory disease and pyometra groups compared with controls. Serum CRP concentrations were markedly increased in both disease groups, reflecting systemic inflammatory responses. In addition, serum albumin concentrations were significantly decreased in diseased dogs compared with healthy controls. Most other biochemical variables, including glucose, creatinine, blood urea nitrogen, total protein, and liver enzyme activities, remained within reference ranges across groups. No statistically significant differences were observed between the non-reproductive inflammatory disease and pyometra groups for any clinicopathologic variable.

### 3.2. Exploratory Overview of Circulating miRNA Expression Patterns

**Figure 1:**
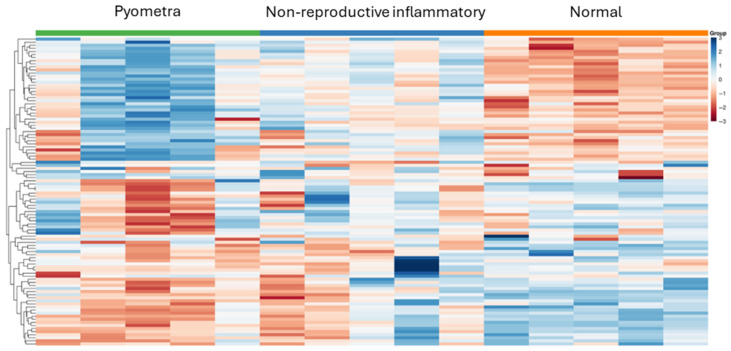
**Exploratory heatmap of circulating miRNA expression patterns across study groups.** Heatmap depicting the relative expression patterns of circulating miRNAs across dogs with pyometra, non-reproductive inflammatory diseases, and healthy controls. miRNAs were selected as the top 100 most variable features across all samples based on variance. Variance-stabilized (VST) expression values were used for visualization and were row-wise scaled (Z-score normalized) to emphasize relative expression patterns across samples. Hierarchical clustering was applied only to miRNAs to identify co-expression patterns, while samples were displayed according to predefined clinical groups due to the limited sample size. The heatmap demonstrates separation between healthy controls and diseased dogs.

Global expression patterns of circulating miRNAs across the three groups are shown in Figure 1 (Figure S1). Heatmap visualization based on variance-stabilized (VST) expression data of the top 100 most variable miRNAs showed separation between healthy controls and diseased dogs. Samples from the non-reproductive inflammatory disease and pyometra groups exhibited expression patterns that differed from those of healthy controls. Although partial overlap between the two disease groups was observed, differences between groups were also evident.

### 3.3. Identification of Condition-Specific and Shared Differentially Expressed Circulating miRNAs

Differential expression analysis was performed by comparing dogs with pyometra and dogs with non-reproductive inflammatory disease separately with healthy controls. The numbers differ depending on the filtering criteria used. Supplementary Figure S2A shows scatter plots of circulating miRNA expression based on DESeq2 results, illustrating the global distribution of miRNAs in both disease groups relative to controls. Red dots represent significantly upregulated miRNAs, whereas green dots indicate significantly downregulated miRNAs compared with healthy dogs. In the pyometra group, 69 miRNAs were upregulated and 53 were downregulated compared with healthy controls. In the non-reproductive inflammatory disease group, 27 miRNAs were upregulated and 16 were downregulated.

**Figure 2:**
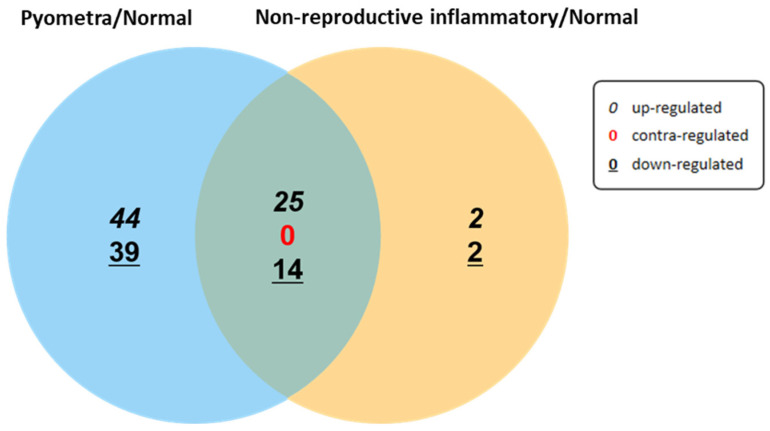
**Venn diagram of condition-specific and shared differentially expressed circulating miRNAs.** Venn diagram illustrating the overlap of differentially expressed circulating miRNAs identified in dogs with pyometra and dogs with non-reproductive inflammatory diseases, each compared with healthy controls. Differential expression was defined using DESeq2 with an adjusted *p*-value (padj) < 0.05 and an absolute log2 fold change (|log2FC|) ≥ 1. The overlapping region represents shared differentially expressed miRNAs, whereas the non-overlapping regions indicate pyometra group and non-reproductive inflammatory disease group differentially expressed miRNAs, respectively. Numbers denote the counts of up-regulated, down-regulated, and oppositely regulated miRNAs in each category.

The identified miRNAs were categorized as pyometra group, non-reproductive inflammatory disease group, or shared between the two disease groups based on overlap across comparisons. As shown in Table 3 and Figure 2, a total of 39 miRNAs were commonly altered in both disease groups compared with healthy controls. Among these, 25 miRNAs were upregulated and 14 were downregulated, and no miRNAs showed opposite directions of regulation between groups. In addition, 83 miRNAs were identified as pyometra-associated and 4 miRNAs as non-reproductive inflammatory disease groups.

Supplementary Figure S2B illustrates the individual expression patterns of the top 30 differentially expressed circulating miRNAs ranked by adjusted *p*-value (padj). Dot plots display expression values for each individual animal, allowing visualization of inter-individual variability as well as overall consistency within clinical groups.

### 3.4. Individual-Level Expression Differences Between Pyometra and Non-Reproductive Inflammatory Disease Group

**Figure 3:**
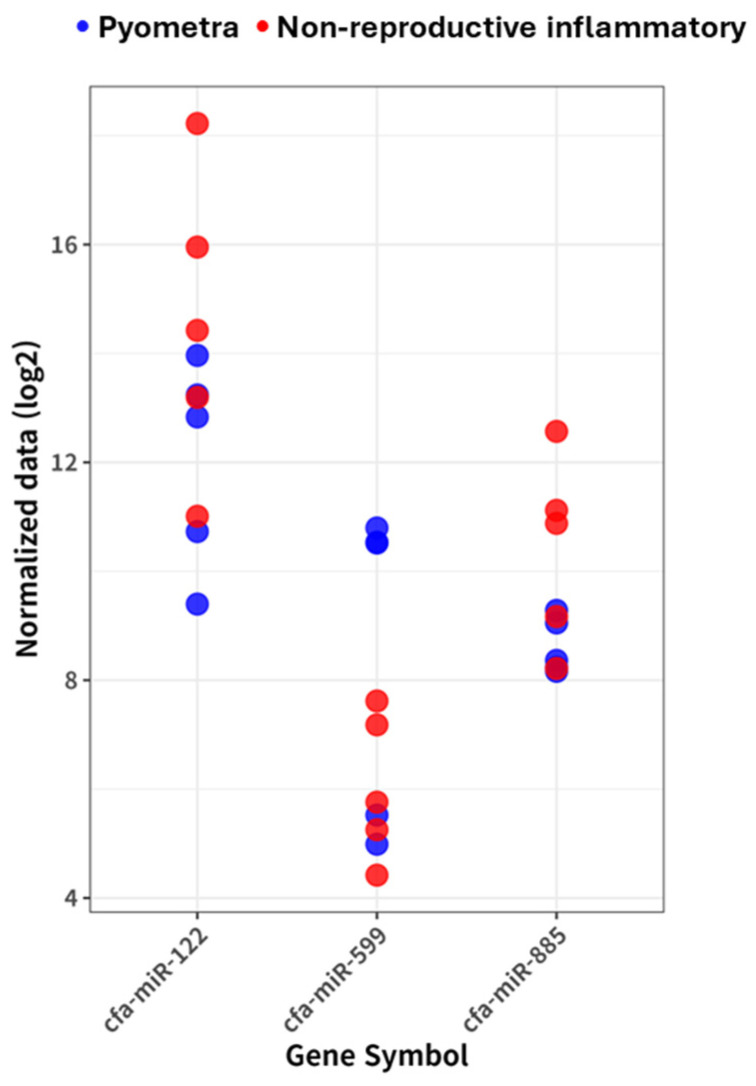
**Individual-level expression of miRNAs differentially expressed between pyometra and non-reproductive inflammatory diseases.** Dot plots showing log2-transformed normalized counts obtained from DESeq2 for selected circulating miRNAs that were significantly differentially expressed between dogs with pyometra and those with non-reproductive inflammatory diseases. miRNAs were identified using DESeq2 with an adjusted *p*-value (padj) < 0.05 and an absolute log2 fold change (|log2FC|) ≥ 1. Each dot represents an individual animal, with blue dots indicating dogs with pyometra and red dots indicating dogs with non-reproductive inflammatory diseases. This figure illustrates individual-level expression variability and highlights quantitative differences between the two disease groups.

Direct comparison between the pyometra and non-reproductive inflammatory disease groups was performed using DESeq2 with an adjusted *p*-value (padj) < 0.05 and an absolute log2 fold change (|log2FC|) ≥ 1 as significance threshold. As shown in Figure 3, three circulating miRNAs, cfa-miR-885, cfa-miR-599, and cfa-miR-122, were identified as significantly differentially expressed between the two disease groups. Among these, cfa-miR-599 showed higher expression levels in the pyometra group compared with the non-reproductive inflammatory disease group, whereas cfa-miR-122 and cfa-miR-885 showed lower expression. Dot plots of these miRNAs illustrated individual-level expression variability while showing overall differences between the pyometra and non-reproductive inflammatory disease groups.

## 4. Discussion

In this study, circulating miRNA expression profiles were characterized in dogs with pyometra, non-reproductive inflammatory diseases, and healthy controls using small RNA sequencing. The analysis revealed both shared inflammatory signatures and condition-specific expression patterns, providing new insight into circulating miRNA alterations associated with naturally occurring canine inflammatory diseases.

Global expression analyses, including heatmap visualization and PCA, demonstrated separation between healthy and diseased dogs. Both disease groups met systemic inflammatory response syndrome (SIRS) criteria and exhibited elevated inflammatory markers such as CRP and leukocytosis. Considering the heterogeneous composition of the non-reproductive inflammatory disease group, which was designed to encompass diverse inflammatory conditions, the observed expression patterns may primarily reflect systemic inflammatory status rather than organ-specific disease involvement.

Scatter plot analysis further demonstrated broad alterations in circulating miRNA expression in diseased dogs compared with healthy controls. A large number of miRNAs showed differential expression in both disease groups, suggesting that systemic inflammatory states are associated with widespread regulatory changes in circulating miRNA expression.

To further characterize these alterations, differentially expressed miRNAs were categorized as shared, pyometra-associated, or non-reproductive inflammatory disease-associated based on overlap between comparisons with healthy controls. Shared miRNAs likely represent regulatory changes associated with systemic inflammation present in both disease groups. In contrast, pyometra-associated miRNAs may reflect biological processes beyond generalized inflammation, including hormonal influences, bacterial infection, and endometrial alterations. Canine pyometra is considered a multifactorial disorder involving a progesterone-dominated endocrine environment, ascending bacterial infection—most commonly *Escherichia coli*—and pre-existing endometrial alterations [9]. In this context, some of the miRNAs identified in the present study may be associated with regulatory mechanisms related to uterine inflammation or tissue remodeling. Conversely, non-reproductive inflammatory disease group miRNAs may reflect regulatory pathways involved in systemic or multi-organ inflammatory processes unrelated to the reproductive system.

Although several circulating miRNAs showed statistically significant differences at the group level, individual expression patterns demonstrated some degree of variability. Such variability is expected in studies involving client-owned dogs, where differences in genetic background, environmental exposure, and disease stage are unavoidable. Previous studies have also highlighted biological variability as a common feature in circulating miRNA profiling across clinical populations [5]. Therefore, the miRNAs identified in the present study should be interpreted as candidate biomarkers that require validation in larger and independent cohorts.

Direct comparison between the pyometra and non-reproductive inflammatory disease groups revealed three differentially expressed miRNAs: cfa-miR-885, cfa-miR-599, and cfa-miR-122. These miRNAs are of particular interest because they were identified as pyometra-associated miRNAs and, when compared separately with healthy controls and non-reproductive inflammatory disease groups, consistently exhibited the same direction of upregulation or downregulation in the pyometra group. Previous studies suggest that these miRNAs are involved in the regulation of inflammatory and immune-related signaling pathways.

MiR-599 has been reported to exert anti-inflammatory effects by targeting ROCK1 and suppressing the JAK2/STAT3 signaling pathway, thereby reducing cytokine production in LPS-induced inflammatory conditions [17]. Accordingly, the increased expression of cfa-miR-599 observed in the pyometra group may reflect a compensatory regulatory response to excessive inflammation. Such upregulation may represent an attempt to counterbalance the intense infection-associated inflammatory response observed in pyometra. miR-885-5p has also been reported to be involved in the amplification of inflammatory responses by repression of HMBOX1, thereby activating NF-κB signaling and promoting inflammasome-mediated pyroptosis [18]. The decreased expression of cfa-miR-885, which corresponds to miR-885-5p, observed in this study may reflect altered or suppressed activation of specific pro-inflammatory and cell death pathways. miR-122 has been reported to be involved in the modulation of inflammatory responses. Previous studies have suggested that miR-122 is associated with the regulation of NF-κB signaling and cytokine production, indicating a potential anti-inflammatory or protective function [19]. Decreased expression of cfa-miR-122 observed in this study may reflect impaired anti-inflammatory regulatory mechanisms, potentially contributing to dysregulated inflammatory responses. Taken together, the overall expression pattern of decreased cfa-miR-885 and cfa-miR-122, along with increased cfa-miR-599, may indicate a shift toward dysregulated and amplified inflammatory signaling, rather than a balanced immune response. This difference in expression between the pyometra and non-reproductive inflammatory disease groups may reflect qualitative differences in inflammatory stimuli rather than disease-specific effects.

Several limitations of this study should be acknowledged. First, the relatively small sample size may limit statistical power and the generalizability of the observed circulating miRNA expression patterns. Second, the non-reproductive inflammatory disease group consisted of heterogeneous diseases affecting multiple organs, which may have contributed to biological variability and potentially confounding effects. This heterogeneity also reflects the complexity of naturally occurring inflammatory conditions in clinical settings. Notably, all included dogs met the criteria for SIRS and exhibited elevated inflammatory markers, such as CRP and leukocytosis, suggesting a shared systemic inflammatory state. Third, bacterial culture was not performed in all dogs with pyometra; therefore, the specific causative pathogens could not be identified, which may limit the interpretation of associations between circulating miRNA profiles and underlying infectious agents. Fourth, differences in reproductive status between groups could not be completely controlled because the pyometra group consisted of intact females, whereas the non-reproductive inflammatory disease and control groups included neutered dogs. Fifth, variability in sample handling, RNA isolation procedures, quantification platforms, and storage conditions, including interim storage at −20 °C prior to −80 °C— may influence circulating miRNA measurements, and the cellular origin and temporal dynamics of circulating miRNAs remain incompletely understood. In addition, hemolysis was assessed visually during sample processing, and no apparent hemolysis was observed. However, quantitative assessment of hemolysis was not performed, which may represent a limitation. The absence of exogenous spike-in controls may have limited the ability to account for technical variability during RNA extraction and quantification. Sixth, RT-qPCR validation of the identified key miRNAs was not performed in this study. As sequencing-based approaches may be subject to technical variability and potential bias, the lack of independent validation may limit the robustness and reproducibility of the findings. Therefore, the identified miRNAs should be interpreted as preliminary and hypothesis-generating candidates, and further validation using RT-qPCR in independent cohorts is warranted. Seventh, diagnostic performance analyses, such as receiver operating characteristic (ROC) curve evaluation and comparison with established biomarkers, were not performed in this study. Therefore, the clinical utility of the identified miRNAs as diagnostic biomarkers remains to be determined. Finally, functional roles of the identified miRNAs were inferred from studies in other species or experimental models, and direct mechanistic validation in canine tissues was beyond the scope of this study.

Despite these limitations, the present study provides exploratory evidence that circulating miRNA profiles in dogs with pyometra and non-reproductive inflammatory disease reflect both shared inflammatory signatures and disease-context–dependent regulatory alterations. These findings support further investigation of circulating miRNAs as potential biomarkers and molecular indicators of inflammatory disease processes in dogs.

## 5. Conclusions

In conclusion, circulating miRNA profiling using small RNA sequencing revealed both shared inflammatory signatures and condition-associated expression patterns in dogs with pyometra and non-reproductive inflammatory diseases. Several miRNAs were commonly altered in both disease groups, suggesting that these shared changes reflect systemic inflammation–associated alterations in circulating miRNA expression. In contrast, miRNAs differentially expressed in pyometra may reflect biological processes beyond generalized inflammation, including hormonal influences, bacterial infection, and endometrial alterations. However, the differences observed between the two disease groups may also be explained by qualitative differences in inflammatory stimuli rather than disease-specific effects. These findings provide preliminary insight into circulating miRNA alterations in naturally occurring canine inflammatory diseases. Further studies with larger cohorts and functional validation are required to clarify the biological roles of these miRNAs and to evaluate their potential as diagnostic or prognostic biomarkers in canine inflammatory disorders.

## Figures and Tables

**Table 1:** Baseline characteristics of dogs are included in the study. Data is presented as mean ± standard deviation for body weight and as median (range) for age and physiological variables unless otherwise indicated. Breed and body condition score are presented as number of dogs. Reference ranges for physiological variables are provided for comparison.

Variables	Reference Ranges	Group		
		Control (*n* = 5)	Non-Reproductive Inflammatory Disease (*n* = 5)	Pyometra (*n* = 5)
Breed	NA	Chihuahua (2), Pomeranian (1), Mixed (2)	Poodle (1), Bichon Frise (1), Bedlington Terrier (1), Maltese (1), Mixed (1)	Dachshund (1), Poodle (1), Mixed (3)
Sex	NA	CM (1), SF (4)	CM (3), SF (2),	F (5)
Weight (kg)		5.76 ± 1.27	7 ± 2.95	4.05± 2.6
Age, years	NA	7 (4–8)	9 (7–16)	10 (5–12)
Temperature	38.3–39.2	38.5 (38.0–38.6)	39.9 (38.3–40.8)	39 (38.9–39.6)
Pulse	70–120	144 (120–150)	150 (120–150)	132 (120–150)
Respiratory rate	18–34	24 (24–30)	24 (20–36)	24 (20–30)
Systolic blood pressure	90–140	120 (120–150)	130 (120–150)	120 (120–150)
Body condition score	1–9	4 (2), 5 (2), 6 (1)	4 (4), 6 (1)	4 (4), 5 (1)
Inflammation-driven diseases	NA	Not applicable	Enteritis (2), pancreatitis (2), bite wound (1)	Pyometra (5)
Concurrent disease	NA	Not applicable	GB sludge (1), MMVD (1)	GB sludge (2)

Abbreviations: NA, not applicable; CM, castrated male; SF, spayed female; F, intact female; GB sludge, gallbladder sludge; MMVD, myxomatous mitral valve disease.

**Table 2:** Clinicopathologic variables in dogs with pyometra, non-reproductive inflammatory diseases, and healthy controls. Data are presented as median (range). Reference ranges are provided for comparison. * Indicates a statistically significant difference compared with the healthy control group (* *p* < 0.05 and ** *p* < 0.01). No significant differences were observed between the non-reproductive inflammatory disease and pyometra groups. Abbreviations: WBC, white blood cell count; HCT, hematocrit; PLT, platelet count; CRP, C-reactive protein; GLU, glucose; CREA, creatinine; BUN, blood urea nitrogen; TP, total protein; Alb, albumin; Glob, globulin; ALT, alanine aminotransferase; ALKP, alkaline phosphatase.

Parameter	Reference Range	Group		
		Healthy Control (*n* = 5)	Non-Reproductive Inflammatory Disease (*n* = 5)	Pyometra (*n* = 5)
WBC (×10^3^/µL)	5.05–16.76	8.00 (5.50–11.31)	19.32 (17.47–38.94)	59.33 (28.96–66.31) **
Neutrophils	2.95–11.64	4.61 (3.11–9.00)	17.16 (10.99–28.97)	26.12 (17.44–39.62) **
Monocytes	0.16–1.12	0.50 (0.23–0.92)	1.35 (0.84–4.28)	13.20 (2.55–21.52) **
Lymphocytes	1.05–5.10	2.48 (1.23–3.54)	2.37 (1.23–5.56)	10.99 (4.86–19.18)
HCT (%)	37.3–61.7	53.9 (53.0–56.2)	38.4 (33.4–61.7)	30.4 (24.2–33.0) **
PLT (×10^3^/µL)	148–484	261 (217–287)	315 (231–716)	275 (111–335)
CRP (mg/dL)	0–1	0.3 (0.1–0.6)	9.6 (6.9–10.0) *	9.7 (7.4–10.0) *
GLU (mg/dL)	74–143	95 (94–120)	93 (66–140)	91 (86–103)
CREA (mg/dL)	0.5–1.8	0.8 (0.7–1.0)	0.7 (0.6–0.9)	0.6 (0.3–1.8)
BUN (mg/dL)	7–27	9 (4–17)	11 (10–24)	20 (9–22)
TP (g/dL)	5.2–8.2	6.4 (6.1–7.1)	6.2 (6.0–7.9)	6.8 (5.8–8.8)
Alb (g/dL)	2.3–4.0	3.4 (3.1–3.7)	2.8 (2.6–3.6)	2.7 (2.2–2.9) *
Glob (g/dL)	2.5–4.5	3.1 (2.4–3.4)	3.4 (3.0–4.6)	4.0 (3.0–6.6)
ALT (U/L)	10–125	74 (33–87)	81 (52–92)	26.0 (13–66)
ALKP (U/L)	23–212	66 (31–97)	268 (66–421)	243 (59–503)

**Table 3:** **Disease-associated and shared differentially expressed circulating miRNAs.** Differentially expressed circulating miRNAs were identified using DESeq2 with an adjusted *p*-value (padj) < 0.05 and an absolute log2 fold change (|log2FC|) ≥ 1. miRNAs were categorized as pyometra-associated, shared, or non-reproductive inflammatory diseaes-associated based on overlap between comparisons and further classified according to direction of regulation.

Group	Regulation	Circulating miRNAs
Pyometra-associated differentially expressed miRNAs	Up regulated (*n* = 44)	cfa-miR-6529, cfa-miR-21, cfa-miR-1307, cfa-miR-1839, cfa-miR-30a, cfa-miR-20a, cfa-miR-590, cfa-miR-24, cfa-miR-8859a, cfa-miR-181d, cfa-miR-328, cfa-miR-101, cfa-miR-20b, cfa-miR-8859b, cfa-miR-148a, cfa-miR-30b, cfa-miR-196b, cfa-miR-7180, cfa-miR-18a, cfa-miR-500, cfa-miR-106a, cfa-miR-425, cfa-miR-362, cfa-miR-660, cfa-miR-148b, cfa-miR-340, cfa-miR-17, cfa-miR-551b, cfa-miR-371, cfa-miR-210, cfa-miR-27b, cfa-miR-1296, cfa-miR-671, cfa-miR-33b, cfa-miR-19a, cfa-miR-8884, cfa-miR-188, cfa-miR-138a, cfa-miR-18b, cfa-miR-330, cfa-miR-1835, cfa-miR-33a, cfa-miR-301b, cfa-miR-599
	Down regulated (*n* = 39)	cfa-miR-122, cfa-miR-379, cfa-miR-127, cfa-miR-409, cfa-miR-382, cfa-miR-494, cfa-miR-299, cfa-miR-329b, cfa-miR-206, cfa-miR-193b, cfa-miR-323, cfa-miR-1, cfa-miR-380, cfa-miR-376a, cfa-miR-217, cfa-miR-376b, cfa-miR-146a, cfa-miR-885, cfa-miR-495, cfa-miR-487b, cfa-miR-802, cfa-miR-107, cfa-miR-10b, cfa-miR-455, cfa-miR-486, cfa-miR-23b, cfa-miR-486-3p, cfa-miR-133c, cfa-miR-410, cfa-miR-342, cfa-miR-125a, cfa-miR-95, cfa-miR-192, cfa-miR-133b, cfa-miR-99b, cfa-miR-103, cfa-miR-195, cfa-miR-452, cfa-miR-125b
Shared differentially expressed miRNAs	Up regulated (*n* = 25)	cfa-miR-30d, cfa-miR-19b, cfa-miR-652, cfa-miR-499, cfa-miR-324, cfa-miR-331, cfa-miR-502, cfa-miR-140, cfa-miR-218, cfa-miR-363, cfa-miR-1343, cfa-miR-1842, cfa-miR-106b, cfa-miR-301a, cfa-miR-450b, cfa-miR-223, cfa-miR-450a, cfa-miR-582, cfa-miR-424, cfa-miR-202, cfa-miR-503, cfa-miR-147, cfa-miR-129, cfa-miR-542, cfa-miR-138b
	Down regulated (*n* = 14)	cfa-miR-211, cfa-miR-489, cfa-miR-203, cfa-miR-432, cfa-miR-216a, cfa-miR-411, cfa-miR-130a, cfa-miR-196a, cfa-miR-199, cfa-miR-214, cfa-miR-205, cfa-miR-224, cfa-miR-361, cfa-miR-183
Non-reproductive inflammatory disease-associated differentially expressed miRNAs	Up regulated (*n* = 2)	cfa-miR-628, cfa-miR-219-5p
	Down regulated (*n* = 2)	cfa-miR-200b, cfa-miR-190b

## Data Availability

The original contributions presented in the study are included in the article/Supplementary Material; further inquiries can be directed to the corresponding author.

## Supplementary Materials

The following supporting information can be downloaded at https://www.mdpi.com/article/10.3390/vetsci13040387/s1: Figure S1: Three-dimensional principal component analysis of circulating miRNA expression profiles; Figure S2: Global distribution and individual-level variability of circulating miRNA expression.

## Abbreviations

ALP: Alkaline phosphatase; BUN: Blood urea nitrogen; CPM: Counts per million; CRP: C-reactive protein; ERK: Extracellular signal-regulated kinase; HCT: Hematocrit; JAK–STAT: Janus kinase–signal transducer and activator of transcription; miRNA: MicroRNA; MODS: Multiple organ dysfunction syndrome; NF-κB: Nuclear factor kappa B; PCA: Principal component analysis; RT-qPCR: Reverse transcription quantitative polymerase chain reaction; SIRS: Systemic inflammatory response syndrome; SOCS3: Suppressor of cytokine signaling 3; TGF-β: Transforming growth factor beta; TLR4: Toll-like receptor 4; TNF-α: Tumor necrosis factor alpha; TMM: Trimmed mean of M values; WBC: White blood cell.
