# Supplementary figures and images for: Comparative Circulating microRNA Profiling in Dogs with Pyometra and Other Inflammatory Diseases
Source: Vet Sci. 2026 Apr 16;13(4):387. doi: 10.3390/vetsci13040387 (PMC13119974; doi:10.3390/vetsci13040387)

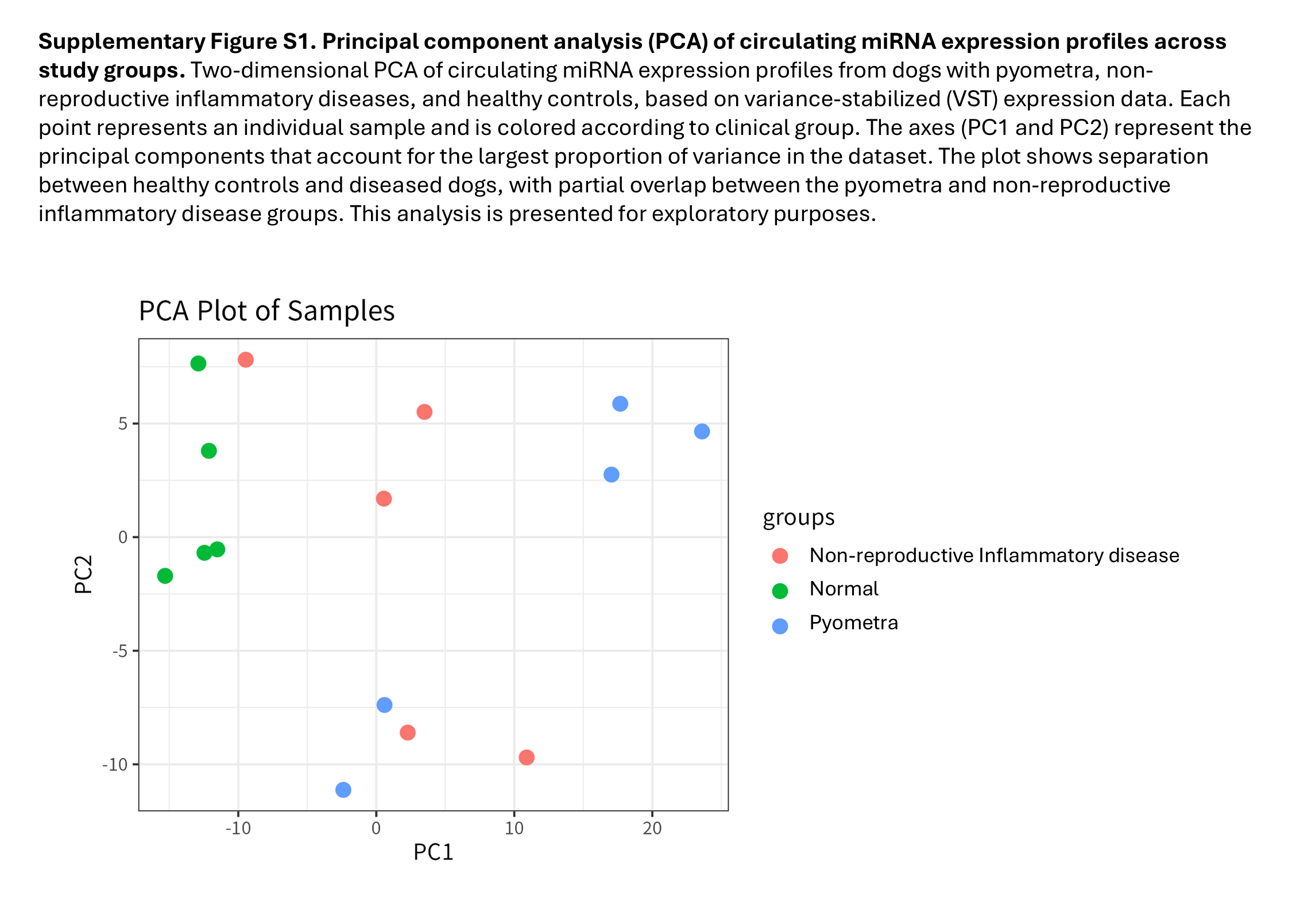

Supplement: Supplementary file 1 [file vetsci-13-00387-s001.zip › Figure S1.TIF]

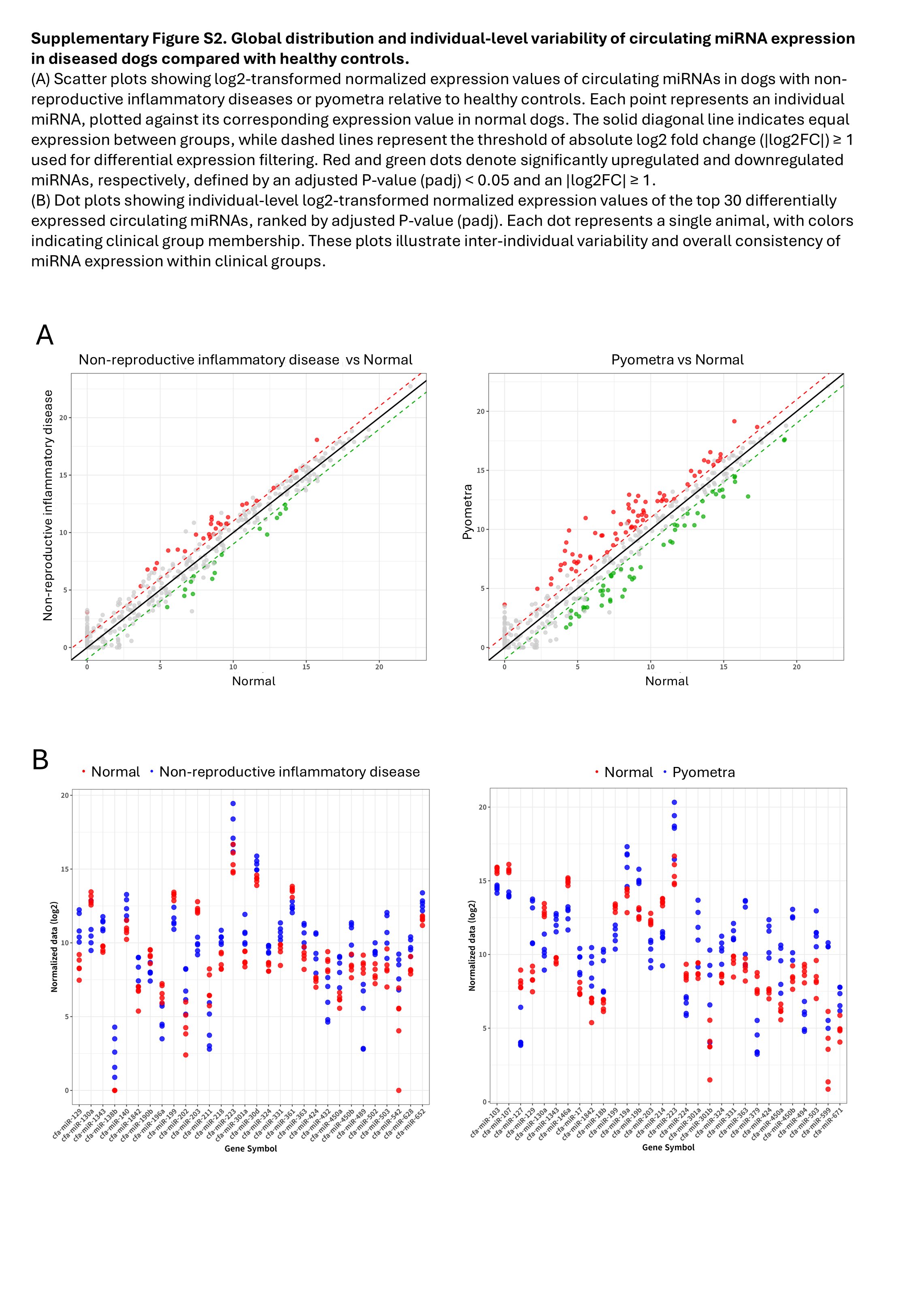

Supplement: Supplementary file 1 [file vetsci-13-00387-s001.zip › Figure S2.TIF]
